# Effect of Ethical Leadership on Nurses' Organizational Silence: The Mediating Role of Organizational Justice

**DOI:** 10.1155/2023/9929435

**Published:** 2023-09-29

**Authors:** Jiachen She, Ruixing Zhang, Yanan Li, Yongxia Mei, Hongfeng Li

**Affiliations:** ^1^School of Nursing and Health, Zhengzhou University, Zhengzhou, Henan 450001, China; ^2^Department of Nursing, The First Affiliated Hospital of Zhengzhou University, Zhengzhou, Henan 450052, China

## Abstract

**Aim:**

The aim of this study is to explore the effects of ethical leadership on nurses' organizational silence and the mediating role of organizational justice.

**Background:**

Organizational silence is considered to be an unethical and destructive influence on healthcare organizations. Ethical leadership has an important influence on nurses' organizational silence. Yet, the effect of mechanism among ethical leadership, nurses' organizational silence, and the perceived organizational justice remains unknown.

**Methods:**

We conducted a cross-sectional survey of 896 nurses in 11 hospitals in Henan Province, China, using a stratified sampling method. Data were analyzed using correlation analysis, one-way analysis of variance, and PROCESS macro.

**Results:**

Chinese nurses spoke highly of the ethical leadership of their nurse managers. Organizational justice and silence among nurses were at moderate levels. Ethical leadership significantly predicted nurses' organizational justice (*β* = 0.453, *P* < 0.001) and nurses' organizational silence (*β* = −0.450, *P* < 0.001). Organizational justice played a partially mediating role in ethical leadership and nurses' organizational silence.

**Conclusion:**

Developing ethical leadership and improving organizational justice for nurses are critical to reduce organizational silence among nurses. *Implications for Nursing Management*. Hospitals can break organizational silence by developing ethical leadership in nurse managers through training related to ethical leadership, such as leadership programs. In addition, nurse managers can reduce the occurrence of organizational silence by increasing nurses' sense of organizational justice.

## 1. Introduction

In the face of multiple challenges in the healthcare environment, healthcare organizations need to innovate and develop steadily, which depends on members actively expressing their opinions or suggestions. The nurse community is an integral part of the healthcare industry and has important speaking rights for patient safety and healthcare quality improvement and has the potential to become the drivers of organizational change and innovation [1]. However, due to various reasons, nurses are more inclined to remain silent when faced with problems, thus missing opportunities to solve problems and leading to the loss of motivation for improvement and innovation in the organization [2, 3]. Organizational silence is a phenomenon in which employees are reluctant to express their views and opinions when confronted with issues within the organization [4]. This silence may be caused by a lack of trust, fear of punishment, or a desire to maintain good relationships and this phenomenon harms nurses' job satisfaction and trust in the organization, while also limiting the organization's innovation capabilities [5, 6]. Organizational silence is considered an unethical behavior that can have a destructive impact on healthcare organizations, often seen as a sign of low employee engagement and commitment [7]. The phenomenon of organizational silence among nurses poses a significant threat to patient safety and the quality of care [8], which makes it crucial to identify and reduce the factors that lead to nurses' silence.

Leadership is defined as “the process by which one person influences a group of people to achieve a common goal” and plays a critical role in the growth and success of an organization [9]. Ethics has always been considered a key element of leadership [10]. Ethical leadership is a new and positive leadership behavior pattern that focuses on the leader's character qualities, values, and ethical behavior, which is defined as leaders demonstrating normatively appropriate behavior through two-way communication, reinforcement, and decision-making [11]. It focuses not only on the achievement of realistic goals but also on influencing the development of employees and the organization sustainably and positively. The value that this leadership style brings is that it builds a high level of trust and belonging, enhances employees' sense of self-worth and intrinsic motivation, and thus promotes the growth and development of both the employee and the organization [12]. According to a systematic review, nurses' work attitudes and behaviors were positively influenced by ethical leadership [13]. Some researchers found that ethical leadership can effectively increase job engagement and enhance voice behaviors [14, 15]. In addition, the related studies in management and psychology fields showed that the role of ethical leadership in breaking organizational silence is crucial [16, 17]. However, how ethical leadership relates to organizational silence and its underlying mechanisms remain under-researched. Therefore, to understand the mechanisms of ethical leadership in depth, further research is required.

The attitudes and organizational behaviors of nurses are closely related to their perception of a fair organizational environment [18]. Organizational justice is defined as how employees perceive fairness in decisions and treatment within the organization [19]. Previous research suggested that an unfair organizational culture may lead to organizational silence [20], while ethical leadership can create a fair atmosphere within the organization [21]. As an important organizational resource, organizational justice can negatively predict employees' organizational silence behavior [6]. In this paper, a conceptual model was proposed which includes the research variable of organizational justice as a mediator between ethical leadership and organizational silence among nurses. In addition, ethical leadership tends to set an example for the ethical norms of the organization and make fair and responsible decisions by balancing various ethical demands [22]. We believed that this leadership style can increase nurses' organizational justice, which in turn can facilitate individual expression and reduce nurses' organizational silence behavior.

Organizational silence is detrimental to effective communication among healthcare professionals and also weakens the organization's learning capacity [23]. In recent years, how to reduce nurses' organizational silence has been a focus of nursing management. Although scholars in other fields have validated that ethical leadership is related to organizational silence [24], the potential mechanisms by which ethical leadership influences organizational silence have not yet been completely known in the field of nursing. It is unclear whether ethical leadership may facilitate nurses' organizational justice and ultimately lessen organizational silence among nurses. Therefore, we conducted this study and examined, first, how ethical leadership, organizational justice, and nurses' organizational silence correlate, and second, whether organizational justice acts as a mediating factor between ethical leadership and nurses' organizational silence.

## 2. Literature Overview

### 2.1. Ethical Leadership and Nurses' Organizational Silence

An ethical leader is a moral example for their followers in the organization, making decisions based on ethical principles and influencing their followers' ethical behavior by communicating with them in a two-way mode, establishing ethical standards, and setting rewards and sanctions [22]. Based on social learning theory, individuals can learn new knowledge, skills, attitudes, and values by imitating a role model's behaviors and qualities [25]. Ethical leadership encourages and supports participation in ethical care practices [26]. And subordinates are encouraged to take part in decision-making, and their opinions are valued [27]. This leadership style inspires employees to learn and emulate behaviors that result in more beneficial behaviors for the organization [21]. In addition, based on the social exchange theory, individuals give positive feedback when they receive trust, respect, and support in interpersonal interactions [28]. Ethical leaders care about respecting their subordinates and creating a safe and supportive environmental climate for them [29], which helps reduce organizational silent behavior among employees. Research in the organizational behavior literature indicates that a negative association exists between ethical leadership and organizational silence [24]. A study by Tak et al. [16] also found that ethical leadership behaviors significantly influence employees' silencing behaviors indirectly by violating the psychological contract, while our study was more concerned with the organizational silence of nurses, we came up with the following hypothesis:

  H1: Ethical leadership is negatively associated with nurses' organizational silence

### 2.2. Ethical Leadership and Nurses' Organizational Justice

Ethical leaders tend to make fair and responsible ethical decisions ethically and are perceived to be honest, upright, and principled in their decision-making [30]. Some findings showed that nurses who are ethical leaders are more likely to engage in interactive justice [21]. In addition, ethical leaders treat others fairly and establish effective ethical standards and a just work environment in the organization [11], which could be an indicator of clues for predicting organizational justice for nurses. Al Halbusi et al. [31] also found that followers of ethical leaders are more likely to perceive more organizational justice and tend to exhibit more workplace ethical behavior. From the above evidence, ethical leadership may shape nurses' organizational justice to some extent. Therefore, we came up with the following hypothesis:

  H2: Ethical leadership is positively related to organizational justice

### 2.3. Nurses' Organizational Justice and Nurses' Organizational Silence

In Colquitt's research, the nurses' perception of organizational justice includes the outcomes achieved (distributive justice), the processes achieved (procedural justice), the relationships established (interpersonal justice), and the information received in the organization (informational justice) [19]. Organizational justice is one of the most important predictors of nurses' work attitudes and behavioral outcomes [18], which has a significant influence on job performance [32], organizational citizenship behavior [33], and work engagement [34], and may be an important variable in organizational silence. There have been many studies confirming the relationship between organizational justice and organizational silence [35, 36]. Gungor and Potuk [36] argued that a decrease in organizational justice arouses an increase in the level of organizational silence. The findings of Korean scholars Kwak and Han [37] also support the negative correlation between organizational justice and organizational silence among nurses. Therefore, we proposed the following hypothesis:

  H3: Nurses' organizational justice is negatively related to nurses' organizational silence

### 2.4. The Mediating Role of Organizational Justice

Based on the social exchange theory, when managers and organizations treat their employees fairly, they will respond with positive behavior based on the principle of reciprocity [38]. Ethical leadership builds good leader-member relationships to enhance teamwork and promote employee voice, thereby reducing the incidence of silence [39]. Conversely, when individuals perceive that they are being treated unfairly, it causes them to engage in negative behaviors such as remaining silent in an attempt to reduce the sense of injustice [20]. A recent study found that ethical leadership behavior influences followers' organizational justice and plays a role in shaping their organizational citizenship behaviors and reducing turnover intentions [40]. However, Wang and Jiang [41] discovered that under the leadership of abusive supervisors, employees lack more opportunities to voice and participate in decision-making and are more likely to remain silent, but organizational justice mediates the effect of abusive leadership on employees' organizational silence. As a result, ethical leadership is more likely to foster a strong sense of organizational justice, which in turn reduces the occurrence of organizational silence among nurses. The following hypothesis was derived from the abovementioned arguments:

  H4: Nurses' organizational justice mediates the relationship between ethical leadership and nurses' organizational silence

**Figure 1:**
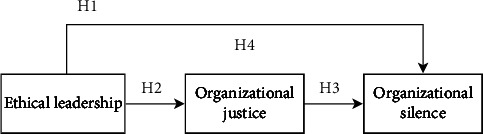
Conceptual model.

A conceptual model of all the hypotheses presented in the study is shown in Figure 1.

## 3. Methods

### 3.1. Design and Sample

A cross-sectional, multicenter study was conducted. It strictly adhered to the guideline for the STROBE statement.

Registered nurses (RNs) recruited were from 11 tertiary general hospitals in the Henan Province of China. Stratified sampling was adopted to select participants. We stratified the 57 tertiary general hospitals in Henan Province according to five geographic divisions and calculated the number of hospitals in each division. At least 1 hospital should be sampled from each division, confirming a sampling ratio of 1/6. A total of 11 hospitals were finally selected from East Henan (1), West Henan (2), South Henan (2), North Henan (3), and Central Henan (3). RNs from different departments in each hospital were randomly selected as study subjects. The inclusion criteria were (a) RNs who had at least one year of work experience and (b) RNs who volunteered to participate in this study. The exclusion criteria were head nurses and new nurses in training. The head nurses are the nurse leaders of the departments and are the subject of the evaluation in the study. The objectivity of the findings will be affected if the head nurses fill in the questionnaires. The new nurses in training have a relatively short time with the head nurses in their departments, which may not be objective enough to evaluate the level of ethical leadership of the head nurses.

**Figure 2:**
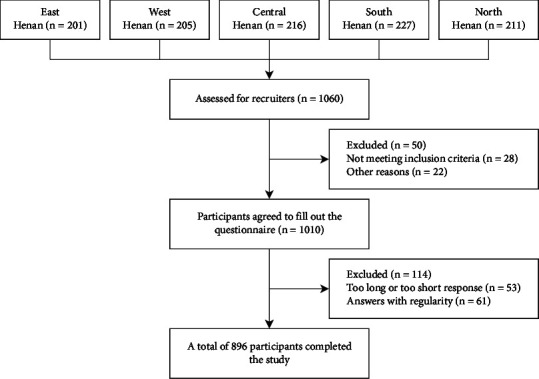
Flowchart on participant recruitment.

The sample size was computed utilizing PASS 15.0 (PASS, Kaysville, UT, USA) (alpha = 0.05; a moderate *f*^2^ effect = 0.15; power = 0.90). The relevant parameters were set based on the results of the preliminary experiment of our study. Considering 20% of invalid samples, a final sample size of 529 cases was estimated. Finally, 896 RNs were recruited. See Figure 2 for the specific recruitment process.

### 3.2. Measurements

#### 3.2.1. General Information Questionnaire

Gender, age, marital status, educational level, professional title, working years, department, monthly income, work area, whether there was clinical teaching experience, whether there was outside study experience, and whether there was scientific research experience (writing papers, applying for or participating in scientific research projects of this specialty, etc.) were all included in the general information questionnaire.

#### 3.2.2. Ethical Leadership at Work Questionnaire (ELW)

ELW is a 38-item survey developed by Kalshoven et al.[27], comprising seven dimensions, namely, people orientation (7 items), fairness (6 items), power sharing (6 items), concern for sustainability (3 items), ethical guidance (7 items), role clarification (5 items), and integrity (4 items). ELW scored ethical leadership on a five-point Likert scale from 1 (strongly disagree) to 5 (strongly agree). A higher score indicates a higher level of ethical leadership. Cronbach's alpha values of the original version of the ELW were 0.90, 0.87, 0.84, 0.84, 0.92, and 0.94. By strictly following Brislin's translation model and conducting cultural adaptation, pretest, and reliability and validity testing, this tool has been translated into Chinese by our team. The Chinese version of ELW contained 37 items in total. Six common factors were identified by exploratory factor analysis, and 59.906% of the variance was contributed by these factors. The Chinese version of ELW has good reliability and validity among nurses. The scale level content validity index/average was 0.970. And Cronbach's alpha was 0.958, the split-half reliability was 0.891, and the test-retest reliability was 0.932. It can be used to assess nurses' perceived ethical leadership level. In our investigation, the overall Cronbach's alpha was 0.977, with each dimension ranging from 0.898 to 0.960.

#### 3.2.3. Organizational Justice Scale

It is a 20 items scale developed by Colquitt [19] and divided into the following four dimensions: distributive justice (4 items), procedural justice (7 items), interpersonal justice (4 items), and informational justice (5 items). The scale employed a 5-point Likert scale, with 1 being strongly disagree and 5 being strongly agree. Higher scores suggested that nurses felt more organizational justice. Cronbach's alpha was between 0.78 and 0.93 in multiple studies covering a variety of fields [42, 43]. And Cronbach's alpha was acceptable in our study, being 0.972.

#### 3.2.4. Organizational Silence Scale

Yang et al. developed the organizational silence scale in 2016 with the Delphi method for nurses [2]. The scale includes 20 items with four dimensions, including defensive silence (6 items), disregard silence (4 items), prosocial silence (4 items), and negative silence (6 items). The scale utilized a 5-point Likert scale, with 1 being the strongest disagreement and 5 being the strongest agreement. The higher the score, the more severe the silent behavior that was taking place. The tool has been demonstrated to be valid and reliable by Chinese researchers, and Cronbach's alpha values were in the range of 0.791 to 0.857. The internal consistency result for our study was 0.969.

### 3.3. Data Collection

An online survey was used to gather the information between March and May 2023. With the consent of the hospital managers, the questionnaires were distributed to each department by the head of the participating hospitals' counterparts. Participants were informed about the research objective and participated voluntarily in the survey. The electronic questionnaire was filled out anonymously, and each question was set as a mandatory question. To guarantee the quality of the return, only one questionnaire could be completed by each IP address.

### 3.4. Ethical Considerations

The Ethics Committee of Zhengzhou University approved this study (ZZUIRB2023-031). At any point during the survey, participants had the option to leave the study. In addition, the purpose of the questionnaire utilized in this study was to gather factual data and information without endangering the individual's physical or mental health.

### 3.5. Data Analysis

IBM SPSS Statistics version 25.0 was used for the statistical analysis of the data. The mean and standard deviation describe the measurements following a normal distribution, and frequency and composition ratio describe counts. Independent samples *t*-test and one-way ANOVA were utilized to describe demographic characteristics and to compare the distribution of nurses' organizational silent behavior. The relationship among ethical leadership, organizational justice, and organizational silence was examined using Pearson's correlation analysis. Finally, the PROCESS macro technique for SPSS was applied to examine the mediation model. Based on 5,000 bootstrap resamples, 95% confidence intervals (CI) for bias correction were calculated. And the presence or absence of mediation effects was judged based on whether the 95% CI contained 0. The model was also controlled for covariates (work area, education, professional title, department, monthly income, and whether there was clinical teaching experience) and standardized for study variables. The test level was *α* = 0.05.

## 4. Results

### 4.1. Sociodemographic Characteristics and Organizational Silence

The survey was completed by 896 registered nurses out of 1,060 participants, for a response rate of 84.53%. There was an overwhelming majority of participants who were female (92.7%), married (79.8%), baccalaureate-educated (84.8%), and had clinical teaching experience (80.9%). More than half of the participants in this study were nurses with professional titles of nurses-in-charge (51.5%) between the ages of 30 and 40 (60.4%). The distribution of sociodemographic characteristics and organizational silence is shown in Table 1.

### 4.2. Study Variable Descriptive Statistics and Correlation Analysis

Table 2 displays the descriptive statistics and the correlations among ethical leadership, organizational justice, and nurses' organizational silence. Compared to the average item score out of 5, the average item for ethical leadership was (4.00 ± 0.62), indicating that the nurses rated nurse managers' ethical leadership as being at a high level; the average item for organizational justice and organizational silence was (3.76 ± 0.75) and (2.35 ± 0.86), respectively, indicating a moderate level of nurses' organizational justice and nurses' organizational silence. Ethical leadership was positively correlated with organizational justice for nurses (*r* = 0.695, *P* < 0.01). And ethical leadership was negatively correlated with organizational silence for nurses (*r* = −0.605, *P* < 0.01). In addition, organizational silence among nurses was inversely connected with their organizational justice (*r* = −0.708, *P* < 0.01).

### 4.3. Single-Factor Analysis of Organizational Silence with Different Demographic Characteristics

According to the independent samples' *t*-test and one-way ANOVA results, the organizational silence scores of participants who had different work areas (*F* = 5.536, *P* < 0.001), education (*F* = 7.076, *P* = 0.001), professional title (*F* = 6.230, *P* < 0.001), department (*F* = 3.14, *P* = 0.003), monthly income (*F* = 3.128, *P* = 0.025), and whether they had clinical experience in teaching (*t* = 2.046, *P* = 0.041) were significant differences (Table 1).

### 4.4. Validation of the Research Hypothesis

**Figure 3:**
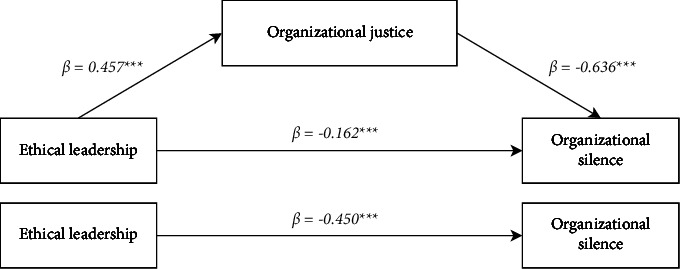
Organizational justice as the mediator between ethical leadership and organizational silence (Andrew Hayes's mediation model 4), ^*∗∗∗*^*P* < 0.001.

Figure 3 and Table 3 show the findings of the significant nature of the direct, indirect, and cumulative effects in the mediation model after controlling for statistically significant demographic variables (work area, education, professional title, department, monthly income, and whether there was clinical teaching experience) as covariates. The overall impact of ethical leadership on organizational silence among nurses was significant (*β* = −0.450, *P* < 0.001), supporting H1. Ethical leadership was positively associated with nurses' sense of organizational justice (*β* = 0.453, *P* < 0.001), supporting H2. Nurses' organizational justice had an opposite association with organizational silence (*β* = −0.636, *P* < 0.001), supporting H3. According to the 5,000 bootstrap resampling findings, ethical leadership through nurses' organizational justice had a significant indirect effect on nurses' organizational silence with 95% CI not including 0 (−0.339 −0.240), supporting H4. Indirect effects accounted for 64% of the total effect of ethical leadership on organizational silence among nurses (indirect effect: −0.288/total effect: −0.450 × 100%).

## 5. Discussion

From the perspective of nurses, this study looked into the connections among ethical leadership, organizational justice, and organizational silence. In particular, we investigated the role of organizational justice as a mediator between ethical leadership and organizational silence among nurses. Our findings provided support for the research hypothesis. Ethical leadership was negatively related to nurses' organizational silence, and organizational justice partially mediated the relationship between ethical leadership and nurses' organizational silence. Our findings also have significant guidelines for nursing management practice.

First, according to the findings, nurse leaders demonstrated high levels of ethical leadership, with mean scores higher than those observed in the study by Awad and Ashour [44]. This study argued that Chinese RNs rated their nurse managers' ethical leadership highly due to the nurse managers' altruistic attitude of caring for and respecting nurses, treating people with fairness and integrity, and acting in a clear manner of responsibility [45]. Numerous studies confirmed the effectiveness of ethical leadership and its correlation with positive outcomes for nurses [13]. And fostering ethical leadership in nursing organizations would help promote nursing professional development [46]. Therefore, it is necessary to encourage nurse managers to adopt an ethical leadership style.

Moreover, according to this study, the mean score of organizational silence among nurses was at moderate levels, which is generally similar to that of other studies on the nurses' organizational silence in China [2, 47], but lower than that of the Philippines' findings of Labrague and De Los Santos [48] and the nurses in Egypt [49]. It may be the result of cultural differences or differences in measurement methods [48]. Our study identified the work area as one of the influencing factors of organizational silence. This may be related to the different levels of economic development in different geographic regions of Henan Province. Central Henan has more tertiary-level hospitals, and tertiary-level hospitals in China pay more attention to the ability of innovation and development [50], giving nurses more voice and participation [1, 47]. In addition, our study showed that nurses with low education levels, low professional titles, lower monthly salaries, and no teaching experience exhibited higher levels of organizational silence. This also suggests that nursing managers can adjust their management strategies in time according to the characteristics of different groups and reduce nurses' organizational silence by focusing on the structural empowerment of low-educated nurses, giving full play to the leading role of nurses with high titles, and constructing a support system for nurses.

Second, as hypothesized by the study, ethical leadership directly and negatively predicted nurses' organizational silence (H1). This finding was consistent with the finding of Li et al. which shows that ethical leadership influenced silent behavior in grassroots' civil servants [17]. Organizational silent behavior means withholding ideas and refusing to share them, which hinders organizational learning and improvement [23] and also increases the intention of the nurse to leave the job [51]. Ethical leadership encourages others to participate in decision-making and action planning by providing a safe and nonthreatening environment to discuss their views and feelings [22, 46]. At the same time, ethical leaders focus on building trust and respect with nurses in two-way communication, which leads to good interpersonal relationships. These behaviors motivate nurses to actively voice their opinions and increased voice behavior and willingness to communicate [15], which reduces the occurrence of organizational silence. Thus, ethical leadership is critical to reduce organizational silence.

Third, the findings confirmed that ethical leadership was positively associated with nurses' organizational justice (H2), with the association between the two having the largest t-value. This result is supported by the related studies in other fields [31]. The relevant literature showed that ethical leadership supports respect for subordinates, follows the principles of fairness and justice in decision-making, ensures positive attitudes of nurses in performing their job duties, and creates a climate of justice [13], which is beneficial to nurses' sense of organizational justice. Therefore, leaders can build a culture of justice in the nursing organization so that every member is treated fairly.

Fourth, according to the results, nurses' organizational justice was negatively associated with nurses' organizational silence (H3), which was in line with those of Liu et al. [52]. High levels of organizational justice boost nurses' commitment to the organization and job satisfaction and promote nurses' psychological wellbeing [18], which will motivate them to actively participate in organizational decisions, build team dialogue and contribute to organizational innovation [3, 6], and avoid indifference to organizational decisions.

Finally, the findings demonstrated that organizational justice acted as a mediating factor between ethical leadership and nurses' organizational silence (H4). Nurses' sense of organizational justice has been demonstrated to be critical to improving the quality of care and job performance in numerous studies [32, 34]. Organizational justice for nurses is a growing area of research, but few studies have provided relevant evidence as to how ethical leadership may affect organizational silence in nursing organizations. Our study shows for the first time that ethical leadership can reduce the occurrence of organizational silence by increasing organizational justice among nurses. This result is easily explained by the fact that ethical leaders who are trustworthy, decent, and principled create a more equitable work environment [11, 21], which helps to increase nurses' sense of organizational justice and reduce their fear of negative reactions, thus motivating nurses to talk about work-related issues and be courageous in expressing their feelings, opinions, and attitudes. According to James et al. [53], nursing leaders who utilize values-based leadership, including ethical leadership, are more likely to engage nurses and encourage them to become involved in the organization, which results in more positive and effective healthcare outcomes. Thus, nurses who have a more positive view of ethical leadership at work mean a greater level of organizational justice and are less inclined to remain silent.

### 5.1. Limitations

There are several limitations. First, all of the samples originated from Henan Province, China, in this study, and the results might not apply to other parts of China or the entire world. In the future, researchers can expand the survey area to collect data representative of nurses nationwide. Second, there was only one source of data for this study, all from nurses' self-reports, potentially subject to the common method bias (CMB). Future research can gather information from a variety of sources, such as nurses, nurse managers, and their colleagues. Third, this study could not use hierarchical linear models (HLM) for statistical analysis because the survey hospitals did not allow us to access unit or hospital department-level information. Finally, being a cross-sectional survey, this study was unable to determine a causal connection between its variables; and it focused solely on how organizational justice acts as a mediator between ethical leadership and organizational silence. To investigate deeper on how ethical leadership affects nurses' organizational silence, further research can use either an experimental or longitudinal design through multiple individual and organizational aspects (e.g., psychological empowerment and organizational climate).

## 6. Conclusions

In this study, the association among ethical leadership, organizational justice, and organizational silence among nurses and the mediating function that organizational justice plays in this was examined. There is a good positive correlation between organizational justice and ethical leadership, and both negatively predict nurses' organizational silence. In addition, the relationship between ethical leadership and nurses' organizational silence is mediated by organizational justice. Therefore, to reduce organizational silence among nurses, nursing managers should focus more on developing ethical leadership and strengthening nurses' sense of organizational justice.

## 7. Implications for Nursing Management

Our findings lead to several implications for nursing management. On the one hand, healthcare organizations can break organizational silence by shaping the ethical leadership style of nurse managers. Training for nurse managers related to ethical leadership, such as an ethical leadership program [54], may help develop ethical leadership. In practicing ethical leadership, nursing managers should focus on empowerment and effective communication with nurses, which allows nurses to gain more autonomy and voice their views on building the organization, thereby breaking organizational silence. On the other hand, establishing a just and fair organizational culture is something that nurse managers should pay special attention to, which will encourage nurses to be actively involved in their work and in turn have the potential to result in less organizational silence, and thus have more opportunities to better nursing care and better health outcomes for patients.

## Figures and Tables

**Table 1:** Participants' demographics and organizational silent distribution (*n* = 896).

Characteristics	*N* (%)	Organizational silence (M ± SD)	*F*/*t*	*P*
*Work area*				
Central Henan	180 (20.1)	46.12 ± 16.73	5.536	<0.001^*∗∗*^
East Henan	189 (21.1)	45.51 ± 17.95		
North Henan	183 (20.4)	45.26 ± 16.12		
South Henan	181 (20.2)	45.75 ± 17.98		
West Henan	163 (18.2)	52.54 ± 15.92		
*Gender*				
Female	831 (92.7)	46.91 ± 17.21	−0.008	0.994
Male	65 (7.3)	46.89 ± 16.70		
*Age*				
<30	238 (26.6)	46.05 ± 16.84	0.417	0.659
30–40	541 (60.4)	47.27 ± 17.37		
>40	117 (13.1)	46.99 ± 16.91		
*Working years*				
<10	360 (40.2)	45.74 ± 17.11	1.476	0.229
10–20	439 (49.0)	47.54 ± 17.21		
>20	97 (10.8)	48.36 ± 17.09		
*Marital status*				
Single	181 (20.2)	47.14 ± 17.46	0.206	0.837
Married	715 (79.8)	46.85 ± 17.10		
*Educational status*				
Diploma	122 (13.6)	41.54 ± 15.30	7.076	0.001^*∗∗*^
Baccalaureate	760 (84.8)	47.72 ± 17.38		
Master	14 (1.6)	49.50 ± 11.91		
*Professional title*				
Primary nurse	92 (10.3)	41.00 ± 14.92	6.230	<0.001^*∗∗*^
Senior nurse	314 (35.0)	46.46 ± 17.80		
Nurses in charge	461 (51.5)	48.70 ± 16.92		
Professor of nursing	29 (3.2)	42.14 ± 15.92		
*Department*				
Critical care	96 (10.7)	47.02 ± 15.90	3.140	0.003^*∗∗*^
Emergency	67 (7.5)	47.87 ± 15.48		
Internal department	250 (27.9)	43.90 ± 17.84		
Obstetrics and gynecology	65 (7.3)	48.71 ± 15.05		
Operation theatre	39 (4.4)	56.23 ± 19.99		
Pediatrics	86 (9.6)	45.72 ± 17.44		
Surgery department	178 (19.9)	47.7 ± 15.76		
Others	115 (12.8)	48.28 ± 18.41		
*Income*				
≤3000	53 (5.9)	41.58 ± 14.70	3.128	0.025^*∗*^
3001∼6000	394 (44.0)	46.09 ± 17.34		
6001∼9000	341 (38.1)	48.60 ± 16.85		
>9000	108 (12.1)	47.16 ± 18.07		
*Teaching experience*				
Teaching	725 (80.9)	47.48 ± 17.41	2.046	0.041^*∗*^
Nonteaching	171 (19.1)	44.50 ± 15.91		
*Study abroad experience*				
Study abroad	342 (38.2)	47.96 ± 16.42	1.449	0.148
Nonstudy abroad	554 (61.8)	46.26 ± 17.58		
*Research experience*				
Research	335 (37.4)	47.79 ± 17.49	1.186	0.236
Nonresearch	561 (62.6)	46.38 ± 16.96		

M, mean; SD, standard deviation. ^*∗*^*P* < 0.05. ^*∗∗*^*P* < 0.01.

**Table 2:** Correlation and descriptive statistics between study variables.

Variable	1	2	Total score (M ± SD)	Average item score (M ± SD)	Cronbach's *α*
(1) Ethical leadership	—	—	148.14 ± 22.92	4.00 ± 0.62	0.977
(2) Organizational justice	0.695^*∗∗*^	—	75.22 ± 15.06	3.76 ± 0.75	0.972
(3) Organizational silence	−0.605^*∗∗*^	−0.708^*∗∗*^	46.91 ± 17.16	2.35 ± 0.86	0.969

M, mean; SD, standard deviation. ^*∗∗*^*P* < 0.01.

**Table 3:** Effect estimates of the hypothesized mode (*n* = 896).

Structural paths	*Coefficient significance*				*95% CI*		*Fitting index*	
	Effect	SE	*t*	*P*	LLCI	ULCI	*R * ^2^	*F*
*Total effect*								
H1: EL ⟶ OS	−0.450	0.020	−22.739	<0.001	−0.489	−0.411	0.384	79.089
*Direct effect*								
H2: EL ⟶ OJ	0.453	0.016	28.887	<0.001	0.423	0.484	0.497	125.295
H3: OJ ⟶ OS	−0.636	0.037	−17.38	<0.001	−0.707	−0.564	0.541	130.426
EL ⟶ OS	−0.162	0.024	−6.796	<0.001	−0.209	−0.115	—	—
*Indirect effect*								
H4: EL ⟶ OJ ⟶ OS	−0.288	0.025^a^	—	—	−0.339	−0.240	—	—
Proportion mediated (EL ⟶ OJ⟶ OS)^b^	64.00%							

*Note*. Controlling covariates: area, educational status, professional title, department, income, and teaching experience. CI, confidence interval; LLCI, lower limit confidence interval; ULCL, upper limit confidence limit interval; SE, standard error; EL, ethical leadership; OS, organizational silence; OJ, organizational justice. ^a^Bootstrap standard error. ^b^The ratio of the indirect effect to the overall effect was referred to as proportion mediation.

## Data Availability

The quantitative data used to support the findings of this study are restricted by the Zhengzhou University Ethics Committee in order to protect participant privacy. Data are available from the corresponding author upon request (lhfchina@zzu.edu.cn) for researchers who meet the criteria for access to confidential data.
